# Anthropogenic Hybridization Contributes to the Naturalization of Introduced Domestic Mallards (*Anas platyrhynchos*) at the Expense of Native New Zealand Gray Ducks (*A. s. superciliosa*)

**DOI:** 10.1002/ece3.71536

**Published:** 2025-06-07

**Authors:** Joshua I. Brown, Jennifer L. Sheppard, Jonathon Mohl, Irene E. Engilis, Andrew Engilis, Philip Lavretsky

**Affiliations:** ^1^ Department of Life, Earth, and Environmental Sciences West Texas A&M University Canyon Texas USA; ^2^ Department of Biological Sciences University of Texas at El Paso El Paso Texas USA; ^3^ University of Aukland and Simax Ecology Tauranga New Zealand; ^4^ Government of Alberta Fort McMurray AB Canada; ^5^ Department of Mathematical Sciences University of Texas at El Paso El Paso Texas USA; ^6^ Museum of Wildlife and Fish Biology, Department of Wildlife, Fish, and Conservation Biology University of California, Davis Davis California USA; ^7^ Department of Wildlife, Fish and Conservation Biology University of California, Davis Davis California USA

**Keywords:** anthropogenic hybridization, Gray Ducks, hybridization, population genomics, waterfowl

## Abstract

Anthropogenically mediated hybridization can lead to several outcomes, with the most severe being hybrid swarms and the genetic extirpation of local populations. With the frequency of introductions by humans increasing, understanding the genetic consequences is critical for future conservation actions. Here, we investigate the consequences of domestic Mallard ( *Anas platyrhynchos* ) introductions in New Zealand (NZ) on the genetic integrity of native NZ Gray Ducks ( *Anas superciliosa superciliosa* ; known locally as Pārera). Although presumed to be genetically extinct, pockets of pure Gray Ducks persist (around 9% of samples), with the western portion of the South Island as the core of their range. In contrast, introduced Mallards have experienced widespread introgression from Gray Ducks that has likely facilitated their rapid establishment and expansion throughout NZ. Moreover, levels of gene flow and divergent selective pressures have resulted in NZ Mallards no longer genetically resembling their original stock and instead have resulted in a hybrid swarm. Estimates of genomic vulnerability for NZ Mallards suggest higher resiliency to future ecological changes as compared to local NZ Gray Ducks. While conservation of NZ's uniquely adapted Gray Duck should remain the priority, we discuss the evolutionary implications of naturalization resulting from anthropogenic hybridization for a now self‐sustaining Mallard population.

## Graphical abstract

Anthropogenic hybridization resulting from the introduction of domestic mallards in New Zealand has led to widespread introgression of native Gray Ducks, resulting in a hybrid swarm. While pure Gray Ducks continue to persist in isolated areas of more undisturbed habitat, mallards have become genetically unique and are likely more resilient to ecological change than the native NZ Gray Ducks.

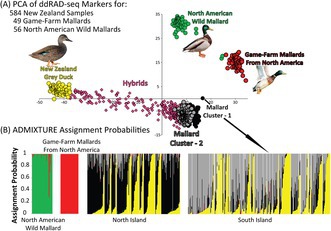

## 1 Introduction

There is growing conservation concern over anthropogenic hybridization, which can occur when previously isolated taxa come into contact as a consequence of human disturbance(s) (Lande 1998; Wayne and Shaffer 2016; Grabenstein and Taylor 2018; McFarlane and Pemberton 2019). Specifically, species that have been historically allopatric (i.e., diverging in non‐overlapping geographic areas) are increasingly experiencing cases of human‐mediated secondary contact (Crispo et al. 2011; Hasselman et al. 2014). This can occur indirectly through range expansion being driven by habitat degradation and/or direct introductions of non‐native species (Crispo et al. 2011; Hasselman et al. 2014). In cases of human introduction, domestic‐origin conspecifics have often been used to supplement existing wild populations, or to establish self‐sustaining populations for recreational purposes (Sheridan 1995; Michaelides et al. 2018). Given that wild and domestic conspecifics are unlikely to have strong barriers to hybridization (Lavretsky et al. 2020), this can lead to introgression from the introduced taxon—i.e., repeated backcrossing that leads to the introduction of novel maladaptive genetic material in the wild populations. When extensive, this can lead to the development of a hybrid swarm, or in extreme cases, the complete loss of genetic integrity (i.e., lineage fusion; Rhymer and Simberloff 1996; Hasselman et al. 2014; Wells et al. 2019). Here, we define a hybrid swarm as a population of individuals from different hybrid backcross generations where significant hybridization has occurred to various degrees with other backcrossed individuals (Rhymer and Simberloff 1996). Such cases are especially problematic for the native species, as locally adapted genotypes and gene combinations can be quickly broken up (Pfaff et al. 2001) or simply overwhelmed by the introduced taxon (Wells et al. 2019; McFarlane et al. 2021). Ultimately, the outcome of hybridization largely depends on the balance between the strength of selection against hybrids and the rate of gene flow between parental groups (Hoskin et al. 2005; Liou and Price 2006). This creates a dynamic system, as these processes are both spatially and temporally heterogeneous (Hoskin et al. 2005; Liou and Price 2006). To fully understand the genomic and adaptive impacts on native and invasive groups, evaluating landscape‐ and genomic‐level sampling data is necessary.

Hybrid swarming generally indicates that reproductive barriers are lacking and that negative selection against hybrids is weak or absent altogether (Gow et al. 2006). In some cases, individuals resulting from hybrid swarms may be able to take advantage of an expanded niche space that was previously maladaptive for either species individually (Glotzbecker et al. 2016). This allows for the introduced taxon to be sustained by the hybrid swarm through the introduction of locally adaptive alleles (Drake 2006). Consequently, locally adaptive alleles from native populations are facilitating the rapid range expansion of the invasive taxon at the cost of their own extirpation (Pfennig et al. 2016). Here, we investigate the outcomes of a putative hybrid swarm consisting of native New Zealand (NZ) Gray Ducks ( *Anas superciliosa superciliosa* ; “Gray Duck,” known by the Māori as Pārera) and recently introduced Mallards ( *Anas platyrhynchos* ) to better understand the costs of anthropogenic hybridization, as well as to determine the process and role that this hybridization has played in facilitating the expansion of the non‐native mallards.

Gray ducks are a subspecies of the Pacific black duck ( *Anas superciliosa* ) and one of fourteen Mallard‐like taxa that comprise the Mallard complex (Lavretsky, McCracken, and Peters 2014). Gray ducks likely colonized NZ from Australia within the last 100,000 years and are largely non‐migratory or nomadic (Rhymer et al. 1994, 2004; Brown et al. 2021). Acclimatization society records from NZ show that starting in the 1860s, greater than 30,000 Mallards were released from a mix of domestic European, domestic North American, and wild North American stocks, with the majority coming from the latter two groups (Williams 1981; Dyer and Williams 2010). Over 80% of releases occurred between 1940 and 1960, by which time Gray Duck habitat had become depleted and populations had dramatically declined (Dyer and Williams 2010). Large releases continued until around 1970, when self‐sustaining domestic Mallard populations were established and began expanding throughout NZ (Caithness et al. 1991; Dyer and Williams 2010; Williams 2017). Today, contemporary estimates of Mallard populations are around three million, while Gray Ducks have been in severe decline (Gillespie 1985; Williams 2017). Mallard × Gray Duck hybrids face no apparent reduction in fertility (Haddon 1984), which has led to extensive hybridization following a century of human‐induced secondary contact (Gillespie 1985; Rhymer et al. 1994, 2004). Despite the historic threat of lineage fusion (Rhymer and Simberloff 1996), it seems that Gray Duck and Mallard population trends have stabilized in the last three decades, with true Gray Ducks making up 5%–10% of NZ's waterfowl populations (Rhymer et al. 2004; Williams 2017). However, both phenotypic characteristics and the limited amount of genetic information used thus far have been unreliable in identifying hybrid backcrosses past the F1 generation (i.e., the crossing of pure parental stock; Williams and Roderick 1973; Braithwaite and Miller 1975; Rhymer et al. 2004). Towards these efforts, we use a landscape‐level sampling effort to determine the true extent of hybridization and whether Gray Ducks persist in NZ.

First, we describe the population structure and geographic distribution of Gray Ducks, Mallards, and hybrids throughout NZ. Given that South Island hybridization rates appear to have stabilized over the last three decades (Gillespie 1985; Rhymer et al. 1994, 2004; Williams 2017), we predict that hybridization may be limited to areas of range overlap between native Gray Ducks and NZ Mallards. This would suggest that geographic barriers (i.e., the Cook Strait and the Southern Alps) and differences in habitat preferences continue to preserve pure Gray Duck populations in areas of more undisturbed native habitat. Next, we use genotype‐environment associations (GEA) to visualize differences in genotypic turnover between Mallards, Gray Ducks, and their hybrids. We hypothesize that models of genotypic turnover would show partitioning between the environmentally adaptive space of Gray Ducks, Mallards, and their hybrids. Alternatively, the introgression of novel alleles from Gray Ducks could increase the Mallard's adaptive potential, facilitating their rapid range expansion throughout NZ. Finally, we modeled GEAs across future projected climate conditions to determine the extent to which changing environmental conditions may affect populations of Gray Ducks and Mallards. We predict that introduced Mallards, as opposed to native Gray Ducks, will be better adapted to expected anthropogenic land‐use changes, as the former has a high affinity for urban and agricultural habitats (Figley and VanDruff 1982).

## 2 Methods

### 2.1 Field Sampling

A total of 584 samples were collected throughout 10 regions in the North and South Islands of NZ from 2014 to 2018 (Table S1). Individuals were putatively assigned to be Gray Ducks, Mallards, or hybrids based on plumage characteristics described by Gillespie (1985); however, we note that all genetic analyses were performed without a priori assignment to groups. Blood was collected from live birds during two concurrent projects, during which all birds were banded to prevent pseudoreplication. This includes (1) 96 samples from the Waikato and Southland regions (Sheppard 2017; Sheppard et al. 2019), and (2) 199 samples collected from five regions of the North Island (including Waikato) during the annual bird banding programs run during January and February by Fish & Game New Zealand Council (FGNZ). Finally, breast or wing muscle tissue was collected from 289 birds salvaged in cooperation with FGNZ during the 2018 hunting season throughout five regions in the South Island (including Southland). Voucher specimens for the 2018 sampling are archived at various biodiversity collections (see Table S1).

All samples were collected in strict accordance with NZ's wildlife and conservation regulations as well as under an approved IACUC protocol (University of Texas at El Paso; Study Number: 967486‐1). Collection was conducted throughout private land under appropriate permits, including scientific collection permits (Te Papa Atawhai, Department of Conservation, Authorization Number: 53520‐FAU), export/import permits (USFWS Permit Number: MB11579C‐2; USDA Permit Number: 132581), and hunting licenses, ensuring compliance with all local and international laws. Collaboration with Fish & Game New Zealand and numerous Fish and Game Councils and landowners was integral throughout the data collection process. Because research and collection occurred solely on private land, Iwi consultation was not required; however, we acknowledge their ancestral presence and, where possible, worked alongside several Māori during the course of the project.

### 2.2 DNA Extraction, ddRAD‐Seq Library Preparation & de‐Multiplexing

Genomic DNA was extracted from blood or tissue using a DNeasy Blood & Tissue kit and following the manufacturer's protocols (Qiagen, Valencia, CA, USA). DNA was visually assessed for quality on a 1% agarose gel and quantified using a Qubit 3 Fluorometer (Invitrogen, Carlsbad, CA, USA) to ensure a minimum concentration of 20 ng/μL. Double digest restriction‐site associated DNA (ddRAD‐seq) libraries were constructed following protocols outlined in DaCosta and Sorenson (2014) also see Lavretsky et al. (2015). In brief, genomic DNA was enzymatically fragmented using SbfI and EcoRI restriction enzymes. Illumina TruSeq‐compatible six‐base‐pair barcodes were ligated to allow for future de‐multiplexing. The barcode‐ligated fragments were then size‐selected for 300–450 bp using gel electrophoresis, followed by gel purification using a MinElute gel extraction kit (Qiagen). We then PCR amplified size‐selected fragments with Phusion high‐fidelity DNA polymerase (Thermo Scientific, Pittsburgh, PA, USA) and purified with a 1.8× solution of AMPure XP magnetic beads (Agencourt, Beverly, MA, USA). Libraries were quantified using a Qubit 3 Fluorometer (Invitrogen, Carlsbad, CA, USA) and pooled in equimolar amounts. Multiplexed libraries were sent to the University of Oregon Core Genomics Facility for 150‐base‐pair, single‐end chemistry sequencing on an Illumina HiSeq 4000. Following sequencing, raw reads were de‐multiplexed based on perfect barcode/index matches using the script *ddRADparser.py* (DaCosta and Sorenson 2014). Raw Illumina sequence reads are deposited in NCBI's Sequence Read Archive (BioProject: PRJNA1238238; PRJNA1106373; PRJNA980669).

Given that Mallard stocks used during introductions in NZ (Williams 1981; Dyer and Williams 2010) came primarily from North American wild and game‐farm lineages, comparable sequence data from previously published wild North American Mallards (*N* = 56) and known game‐farm Mallards (*N* = 49) were included as reference populations to understand their relationship to today's NZ Mallard (BioProject PRJNA591912; Lavretsky et al. 2020). Additionally, while we recognize that a limited number of European Mallards were also used as stock, Lavretsky, Mohl, et al. (2023) have shown high levels of shared ancestry with very little genomic differentiation of North American game‐farm lineages. All 689 raw FASTQ format sequences were trimmed and poor quality sequences discarded using the program trimmomatic v. 0.40 (Bolger et al. 2014). The remaining quality reads were then mapped to the chromosomal‐level reference wild Mallard genome (Lavretsky, Hernández, et al. 2023), using the Burrows Wheeler Aligner v. 07.15 (bwa) (Li and Durbin 2011). Alignments were then sorted, indexed, and genotyped using Samtools v. 1.6. To ensure retention of only high‐quality sequences, we set a base pair PHRED and strand quality scores at ≥ 30 and required a base‐pair sequencing depth of 5× (i.e., 10× per genotype) to be called. The program PGDspider v2.1.1.2 (Excoffier and Lischer 2010) was used to convert VCF files into the FASTA file format. Sequences were then further filtered to remove positions with less than 80% of alleles present using in‐house Python scripts available at https://github.com/jonmohl/PopGen.

Finally, sex was assigned to each sample based on differences in sequencing depth across autosomal and sex chromosome‐linked loci (Lavretsky et al. 2015; Lavretsky, Janzen, and McCracken 2019). Specifically, we expect near‐zero levels of sequencing depth across W‐sex chromosome‐linked loci but near equal depth for Z‐sex chromosome‐linked loci when compared to autosomal loci for the homogametic sex (i.e., males = ZZ). In contrast, we expect to recover about half the sequencing depth at both W‐ and Z‐sex chromosome‐linked loci as compared to autosomal loci for the heterogametic sex (i.e., females = ZW).

### 2.3 Mitochondrial DNA

Primers L78 and H774 were used to sequence 655 bp of the mtDNA control region (Sorenson and Fleischer 1996; Sorenson et al. 1999) following protocols outlined in Lavretsky, Hernández‐Baños, and Peters (2014). Final products were sequenced on an ABI 3730 (Applied Biosystems, Life Technologies, Carlsbad, California, USA) machine at the University of Texas at El Paso BBRC Genomic Analysis Core Facility. We then aligned and edited sequences using Sequencher v. 4.8 (Gene Codes Corporation, Ann Arbor, MI, USA). Sequences were deposited in GenBank (Accession numbers: PP746861—PP746942; PV346099—PV346671; OR89157—OR90543). Note that mtDNA control region sequences for reference North American wild and game‐farm Mallards (Lavretsky, Hernández‐Baños, and Peters 2014; Lavretsky, McCracken, and Peters 2014; Lavretsky, DaCosta, et al. 2019; Lavretsky et al. 2020) were included in subsequent analyses. Structure across mtDNA haplotypes was visualized with a median‐joining haplotype network calculated in the program Network v. 4.5.1.0 (Bandelt et al. 1999).

### 2.4 Nuclear Population Structure and Estimates of Genetic Diversity

Nuclear population structure was based on independent bi‐allelic ddRAD‐seq autosomal single‐nucleotide polymorphisms (SNPs) and without using a priori assignment of individuals to populations or species. First, a custom Python script (Lavretsky et al. 2020) was used to extract bi‐allelic SNPs from a concatenated FASTA file of all autosomal loci. Next, PLINK v. 1.90 (Purcell et al. 2007) was used to filter for singletons (i.e., minimum allele frequency (‐‐maf 0.002)), any SNPs missing ≥ 20% of data across samples (‐‐geno 0.2), as well as any SNPs found to be in linkage disequilibrium (LD) (‐‐indep‐pairwise 2 1 0.5). One of the two SNPs was randomly excluded if an LD correlation factor (*r* ^2^) greater than 0.5 was obtained.

We used Principal Components Analysis (PCA) to visualize the population structure in the R package Adegenet using the ‘*dudi.pca*’ function (Jombart 2008). Next, we estimated assignment probabilities for one through ten populations (K) using the program ADMIXTURE v. 1.3 (Alexander et al. 2009; Alexander and Lange 2011), running 100 iterations of each K. The optimal number of populations (K) was based on the lowest averaged CV‐error across all 100 replicates per K (Zhou et al. 2011). We used the R package PopHelper (Francis 2017) to convert all ADMIXTURE outputs into CLUMPP v. 1.1 (Jakobsson and Rosenberg 2007) input files. Final assignment probabilities were based on the optimal clustering alignment across all 100 replicates per evaluated population K value using the GreedySearch algorithm for 1000 iterations as implemented in CLUMPP v. 1.1. Finally, we used the R package PopGenome (Pfeifer et al. 2014) to estimate relative divergence (Φ_ST_) for concatenated Autosomal and Z‐chromosome ddRAD‐seq loci as well as mtDNA sequences.

Additionally, we used fineRADstructure (Malinsky et al. 2018) to more closely look at shared ancestry among Gray Ducks, NZ Mallards, North American Mallards, and reference game‐farm Mallards. Briefly, fineRADstructure identifies the most recent coalescent events among sample‐by‐sample pairwise comparisons to infer relatedness among individual samples and is informative in cases of recent and ongoing gene flow (Lavretsky, DaCosta, et al. 2019; Brown et al. 2020). Using the same set of bi‐allelic SNPs for fineRADstructure, samples were assigned to populations using 1000,000 iterations of the tree‐building algorithm to assess genetic relationships among clusters. Results were visualized as heat maps using the provided R scripts fineradstructureplot.r and finestructurelibrary.r (available at https://github.com/millanek/fineRADstructure).

### 2.5 Establishing Hybrid Indices

To assign admixed individuals to hybrid or backcross generations, we used bi‐allelic autosomal SNPs and followed the methods outlined in Lavretsky et al. (2016). In short, initial ADMIXTURE assignment probabilities were used to demarcate individuals with ≤ 5% interspecific assignment probability as putatively pure Gray Ducks or NZ Mallards. Ten F1 hybrids were first simulated by randomly sampling alleles from the Gray Duck and Mallard ‘gene pool’ across bi‐allelic SNPs, and randomly sampling each position based on a probability proportional to the allelic frequency in each respective gene pool. Subsequently, five of these F1 hybrids were then each simulated to backcross into either the Gray Duck or Mallard parental gene pool for up to nine generations. In total, 10 independent simulations were run, with outputs subsequently input into ADMIXTURE to estimate assignment probabilities under K population models of 2 and 3. For each K, 25 iterations were run per simulation for a total of 250 ADMIXTURE outputs generated per K, which were then combined and converted in PopHelper (Francis 2017) into CLUMPP input files. Once again, we employed the Large Greedy algorithm and 1000 random permutations, with final admixture proportions for each K and per sample assignment probabilities based on CLUMPP analyses of all 250 replicates per K. Per generation expected assignment probabilities were based on the average of either all ten (F1) or each of the five (F2–F10) backcrosses, along with each lower and upper limit. Empirical ADMIXTURE data was then fit to the simulated hybrid indices in order to calculate the proportion of samples that fell within each hybrid generation. Samples that fell outside the lower and upper limits of each hybrid class were considered generationally unknown, and thus classified as hybrid swarm backcrosses (Lavretsky et al. 2016; Lavretsky, DaCosta, et al. 2019; Lavretsky, Janzen, and McCracken 2019).

### 2.6 Genotype‐Environment Association Modeling With Gradient Forest

We obtained high‐resolution environmental data across NZ from several public databases, with a focus on 27 annual and seasonal environmental variables thought to be related to bird physiology and ecology (Table S2; Hijmans et al. 2005; Brown et al. 2022). This included 19 climate variables from the Worldclim version 1.4 database (Hijmans et al. 2005); Landsat Normalized Difference Vegetation Index (NDVI), Enhanced Vegetation Index (EVI), and Net Primary Productivity data from the USGS AppEEARS database (https://lpdaacsvc.cr.usgs.gov/appeears); and elevation data from the Global Land Cover Facility (http://www.landcover.org). In order to differentiate the effects of annual versus seasonal vegetation processes, we calculated an average annual, winter (June), and summer (December) value for NDVI, EVI, and NPP based on data collected from 2000 to 2019.

Samples were categorized as Gray Ducks or Mallards, and early‐generation hybrids (i.e., F1–F3, including hybrid swarm individuals that fall between those generations) based on the hybrid indices classifications. Note that Gray Ducks and Mallards also included extreme late‐generation backcrosses that had interspecific assignment probabilities of ≤ 5% and 6%, respectively, as these were the thresholds assigned by ADMIXTURE simulations. Following the approach of Bay et al. (2018), also see Brown et al. (2022), we used a GF analysis as implemented in the R package gradientForest for GEA analysis (Ellis et al. 2012). GF analysis was originally created to detect the effects of environmental predictor variables on species turnover across a landscape (Ellis et al. 2012) but has since been adapted for measuring allelic frequency turnover (Fitzpatrick and Keller 2015; Bay et al. 2018; Brown et al. 2022). An additional advantage of these models is that they are generally unaffected by multicollinearity in predictor variables, as predictor variables are randomly withheld during each random forest iteration (Fitzpatrick and Keller 2015; Alvarado et al. 2022).

Following the protocols of Brown et al. (2022), we converted all SNP data into minor allele frequencies and subsequently filtered any SNP that was polymorphic in fewer than five total sampling sites (Fitzpatrick and Keller 2015). To assess the performance of GF models for both species, we compared 100 models created with randomized environmental data to those generated for our empirical data. To visualize GF models across NZ, values for each of the top five environmental variables (based on *R* ^2^ weighted importance) were summarized using a PCA. Finally, these transformed values were used to create an RGB color scale to visualize different patterns of adaptive genetic diversity across the landscape. In the end, changes in color reflect changes in allele frequencies with respect to the environmental predictor variables—i.e., the greater the change in color, the greater the change in allele frequencies for putative environmentally adapted loci. This allows us to draw conclusions about how the environment has affected spatial patterns of genetic diversity and potentially driven local adaptation. Finally, we used the ‘*combinedGradientForest*’ function to combine independent species' models as well as early‐generation hybrid models of allele frequency turnover to identify differences in genetic niche space between species (Brown et al. 2022).

Finally, to estimate effects of climate change on Gray Ducks, as well as the adaptive potential of introduced Mallards and their hybrids, we used GF to model GEA across future climate conditions and subsequently measured the genomic offset (i.e., Euclidean distance between contemporary and future GF models; Bay et al. 2018) from the current GEA space (also see Brown et al. 2022). Future bioclimatic variables from Global Climate Models (GCM) were downloaded at the highest available resolution for two future (2070) scenarios of climate change (rcp2.6 and rcp8.5; CCSM4 at 30 arc‐second resolution; Hijmans et al. 2005).

## 3 Results

After filtering, a total of 3121 ddRAD‐seq loci remained, with 2943 (369,313 bp) and 178 (22,527 bp) being assigned to the autosomal and Z‐sex chromosomes, respectively. An additional 13 loci were assigned to the W‐sex Chromosome (1680 bp). Our dataset consisted of an average median sequencing depth of 141 (range = 25–246) reads per locus per sample, and an average of 98% of alleles present per locus. Plotting sequencing depth ratios between sex and autosomal‐linked loci reliably determined sex across samples (Figure S1). Finally, 600 bp of overlapping mtDNA COI was sequenced for a total of 686 samples (of 689; Table S1).

### 3.1 Population Structure and Hybrid Indices

**FIGURE 1:**
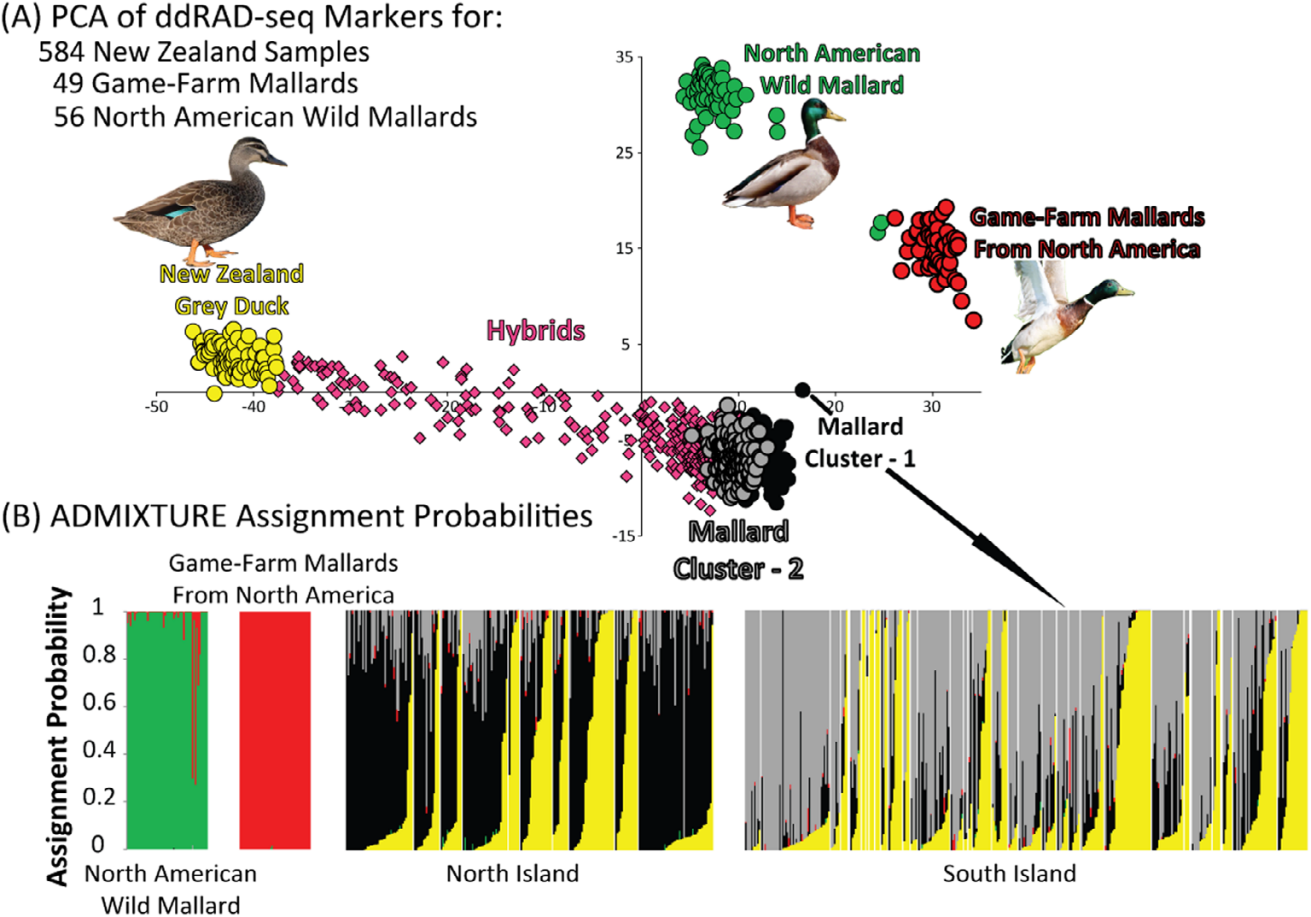
(A) Principal Component Analysis and (B) ADMIXTURE assignment probabilities of 18,882 bi‐allelic ddRAD‐seq nuclear SNPs assayed across 689 samples of Gray Ducks and Mallards from NZ, as well as comparable reference North American wild and game‐farm Mallards.

**FIGURE 2:**
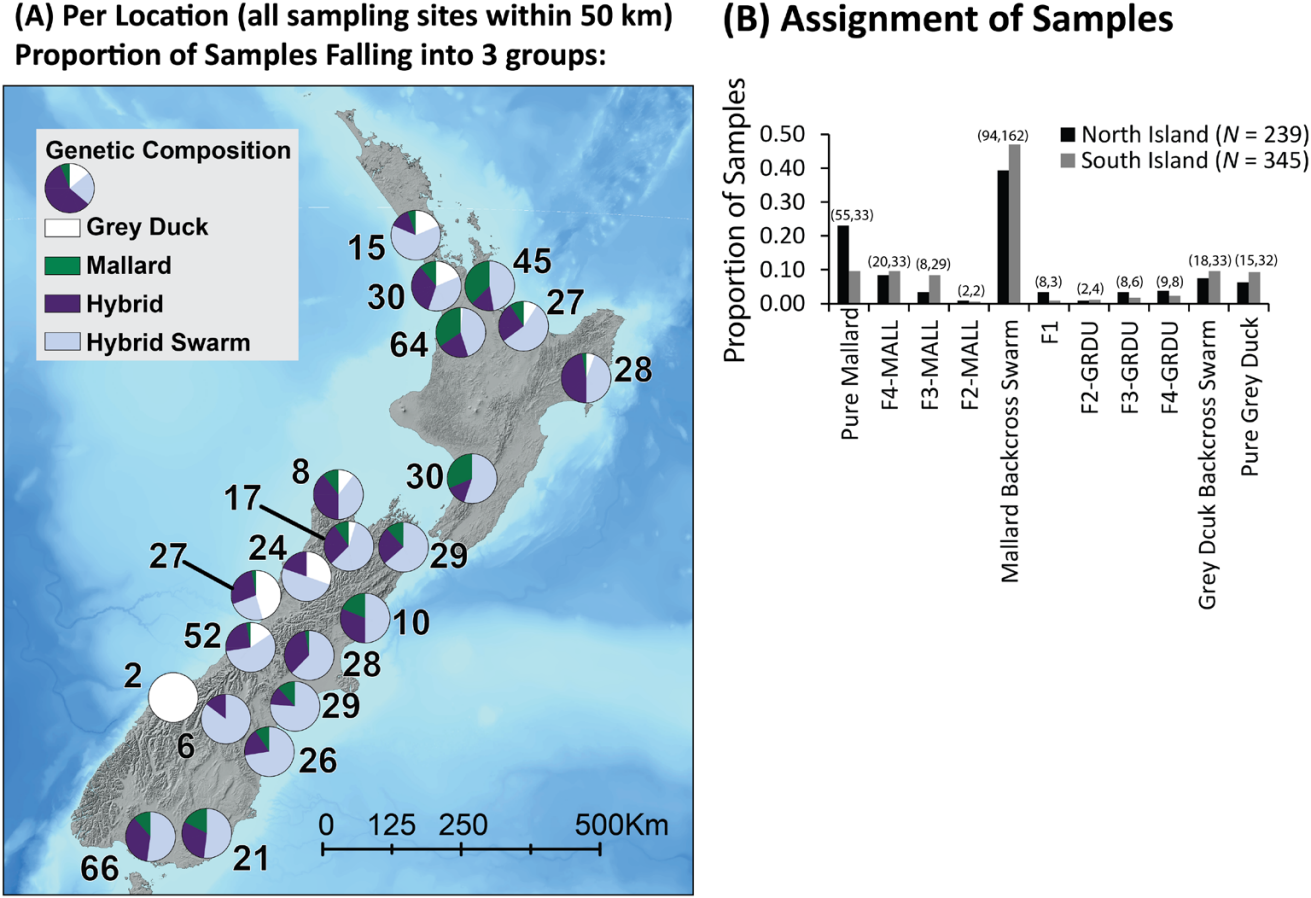
(A) The proportion of samples from each parental and hybrid class mapped across NZ—note that sampling locations within 50 km were grouped. Assignment probability categories were binned based on ADMIXTURE simulated replications of hybridization (F1) and nine generations of backcrossing (F2—F10; see Figure S3 for more information on ADMIXTURE simulations). (B) The total proportion of samples falling into each ADMIXTURE class is summarized by island.

All population structure analyses were based on a dataset of 689 samples and 18,882 bi‐allelic autosomal SNPs that met filtering criteria. A total of five unique genetic clusters were recovered when plotting the first two principal components of PCA and in assignment probabilities estimated in ADMIXTURE based on a K population model of 5; these included: wild North American Mallards, game‐farm Mallards, Gray Ducks, and two clusters of Mallards from NZ corresponding to the North and South Islands, respectively (Figure 1). In addition, a large number of samples represented hybrids as they were identified in intermediate PCA space between Gray Ducks and the two NZ Mallard clusters, as well as assigned to each of these parental clusters in ADMIXTURE analyses (Figure 1). Although NZ Mallards were assigned to one of two unique Mallard genetic clusters, many had small interspecific assignment (i.e., ≤ 5%) to the game‐farm genetic cluster. Unlike NZ's Mallards, however, putative Gray Ducks were assigned to a single genetic cluster without any assignment to reference wild or game‐farm Mallards (Figures 1 and 2A).

Given the variance in interspecific assignment among NZ samples (Figure 1), formal assignment across samples was based on indices derived from simulations of expected assignment probability (Figure S3). For simulations, individuals with ≥ 95% assignment to either Gray Duck or Mallard genetic clusters were used, with the latter being the summation of a sample's assignment to the North and South Island Mallard genetic clusters as recovered in initial ADMIXTURE results (see above); doing so accounts for any potential retained ancestry as well as for small frequencies of introgression (Lavretsky, DaCosta, et al. 2019). At a population model of K = 2, assignment probabilities to Gray Duck or Mallard plateaued at 100% and around 99%, respectively, after six generations; results were statistically similar when analyzing a K population of three (two‐tailed *t*‐test *p* = 0.92; Figure S3; Table S3). Regardless of population model, we report predictable declines in interspecific assignment with each generation of backcrossing (Lavretsky et al. 2016; Lavretsky, Janzen, and McCracken 2019), with individual lineages becoming genetically indistinguishable from their backcrossed population by the third generation of backcrossing (i.e., F4 generation; Table S3). Given the inter‐Island population structure of NZ's Mallards, empirical samples were assigned to backcrossed indices established from a *K* population model of three. Doing so, we recovered 47 genetically pure Gray Ducks and 88 samples of what we now designate here as genetically pure NZ Mallards (Figure 2B; Figure S3). Assignment probabilities of 11 samples were within expectations of an F1 hybrid, and 22% (*N* = 131) of samples were classified within the remaining backcrossed generations (i.e., F2—F4; Figure 2B). The remaining 307 (53%) samples fell between simulated ranges across hybrid indices, suggesting these are the result of hybrid × hybrid pairings from unknown generations, and were therefore considered as part of a hybrid swarm; 84% of hybrid swarm backcrosses were towards NZ Mallards (*N* = 51 Gray Duck backcrosses; *N* = 256 Mallard backcrosses).

**FIGURE 3:**
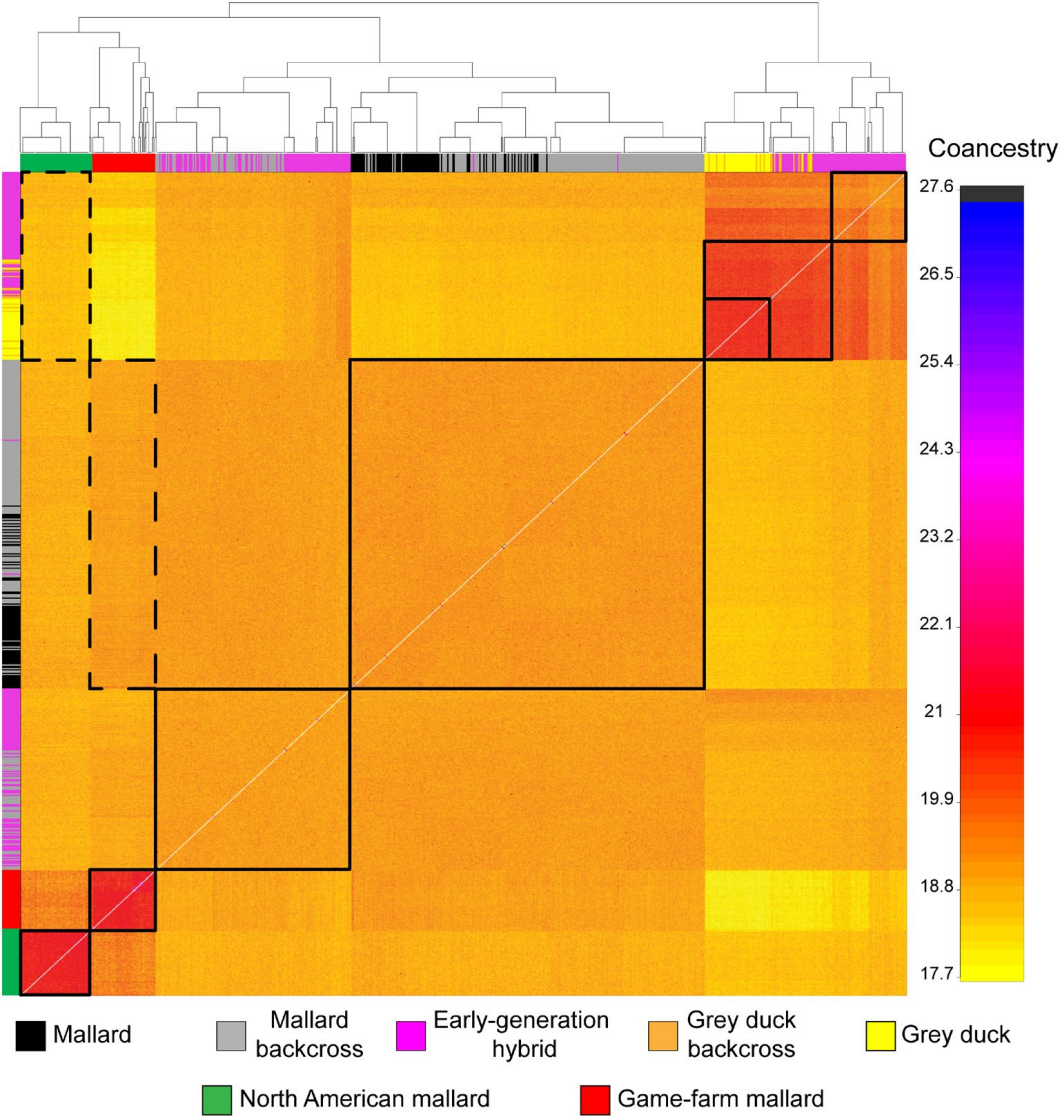
fineRADstructure individual co‐ancestry coefficient matrix estimated using 18,882 bi‐allelic ddRAD‐seq autosomal SNPs. Note that this analysis was based on 698 samples, including reference wild North American and game‐farm Mallards (Table S1). The level of recent co‐ancestry is color coded from low (yellow) to high (blue). We color code Mallards by origin (NZ vs. NA wild vs. game‐farm), as well as identify Gray Duck × Mallard hybrids and hybrid backcrosses.

Estimates of co‐ancestry from fineRADstructure generally corresponded with ADMIXTURE and PCA results, showing five clusters (Figure 3). Individual co‐ancestry within Gray Ducks, NA Mallards, and game‐farm Mallards was similar, suggesting reduced diversity as compared to NZ Mallards. Although early generation hybrids were split (see dendrogram Figure 3), both groups showed nearly equal amounts of co‐ancestry with NZ Mallards and Gray Ducks. Additionally, there was no distinct structure between clusters of pure NZ Mallards and late‐generation backcrosses as previously identified by ADMIXTURE; co‐ancestry between these two groups seems homogeneous. Next, while wild NA Mallards shared similar levels of co‐ancestry with NZ Mallards and Gray Ducks, game‐farm Mallards shared the least amount of co‐ancestry with pure Gray Ducks and late‐generation backcrosses, likely due to NZ Mallards being derived from game‐farm Mallard stock. This leaves NZ Mallards uniquely clustered between Gray Ducks and other reference Mallards (Figure 3), although with a slightly higher affinity to these other reference Mallards. Overall, this indicates significant and ongoing introgression from Gray Ducks.

Finally, pair‐wise population Φ_ST_ estimates were based on the above groupings. Patterns of differentiation were similar across all three marker types (i.e., Autosomal, Z‐chromosome, and mtDNA; Figure S2). Genetically pure Gray Ducks showed very little structure between the North Island (NI) and South Island (SI) (Autosomal Φ_ST_ = 0.0030; Z‐chromosome Φ_ST_ = 0.0010). Relative differentiation between North and South Island for NZ Mallards was 1.2 (Φ_ST_ = 0.0030) and 4.6 (Φ_ST_ = 0.0050) times that of Gray Ducks for Autosomal and Z‐chromosome loci, respectively. Divergence in mtDNA between North and South Island for NZ Mallards (mtDNA Φ_ST_ = 0.019) was only one third that of Gray Ducks (mtDNA Φ_ST_ = 0.061). Relative differentiation in autosomal markers between Gray Ducks and NZ Mallards (Φ_ST_ = 0.11) was only around 0.5 and 0.8 of that with game‐farm (Φ_ST_ = 0.22) and North American (Φ_ST_ = 0.14) Mallards, respectively. Patterns of mtDNA divergence were similar, with Gray Ducks being most strongly differentiated from game‐farm Mallards (mtDNA Φ_ST_ = 0.66) followed by NZ Mallards (mtDNA Φ_ST_ = 0.50).

### 3.2 Mitochondrial DNA Haplotype Structure

The mtDNA haplotype network revealed two known group I and group II haplogroups previously identified in Gray Ducks (Figure S4; Rhymer et al. 1994, 2004; Brown et al. 2021), as well as the known Old World (OW) A and New World (NW) B Mallard haplogroups (Figure S4; Avise et al. 1990; Kulikova et al. 2005; Lavretsky, McCracken, and Peters 2014). Most genetically pure Gray Ducks (87%; 41 of 47) and Gray Duck backcrosses (75%; 100 of 134) contained a Pacific black duck group I or group II haplotype (Figure S4), while Mallards and Mallard‐like samples collected throughout NZ rarely shared a Pacific black duck haplotype (6%; 27 of 462). Of the 18 and 23 unique haplotypes found in each of the groups I and II haplogroups, four and nine were shared between Gray Duck and Mallard‐like samples, respectively. First‐generation hybrids (F1) were split between the group I (*N* = 1), group II (*N* = 3), and OW A (*N* = 7) haplogroups. Interestingly, 11 (2%) NZ Mallards shared a single NW B haplotype, while no Gray Ducks did. The OW A haplogroup contained the majority of NZ Mallard samples (71%; 441 of 572); and despite the presence of 52 unique haplotypes, 69% of these individuals (304 of 441) shared a single haplotype (Figure S4).

### 3.3 Genotype‐Environment Association Modeling

The mean *R* ^2^ value for GF models of Gray Ducks (*R* ^2^ = 0.104; *t‐test*(103) = 4.41, *p* < 0.001), NZ Mallards (*R* ^2^ = 0.105; *t‐test*(100) = 9.87, *p* < 0.001), and early‐generation hybrids (*R* ^2^ = 0.100; *t‐test*(100) = 37.6, *p* < 0.001) all performed better than the randomized datasets. Three of the top five most predictive environmental variables in Gray Ducks were related to seasonal changes (Bio.9, Bio.15; Figure S5), with geography (i.e., Elev) also playing an important role. Conversely, GEAs in Mallards were primarily driven by seasonal temperature variables (Bio.4, Bio.7, Bio.2); however, annual and seasonal vegetation (NPP, NDVI.June) were also important (Figure S6).

**FIGURE 4:**
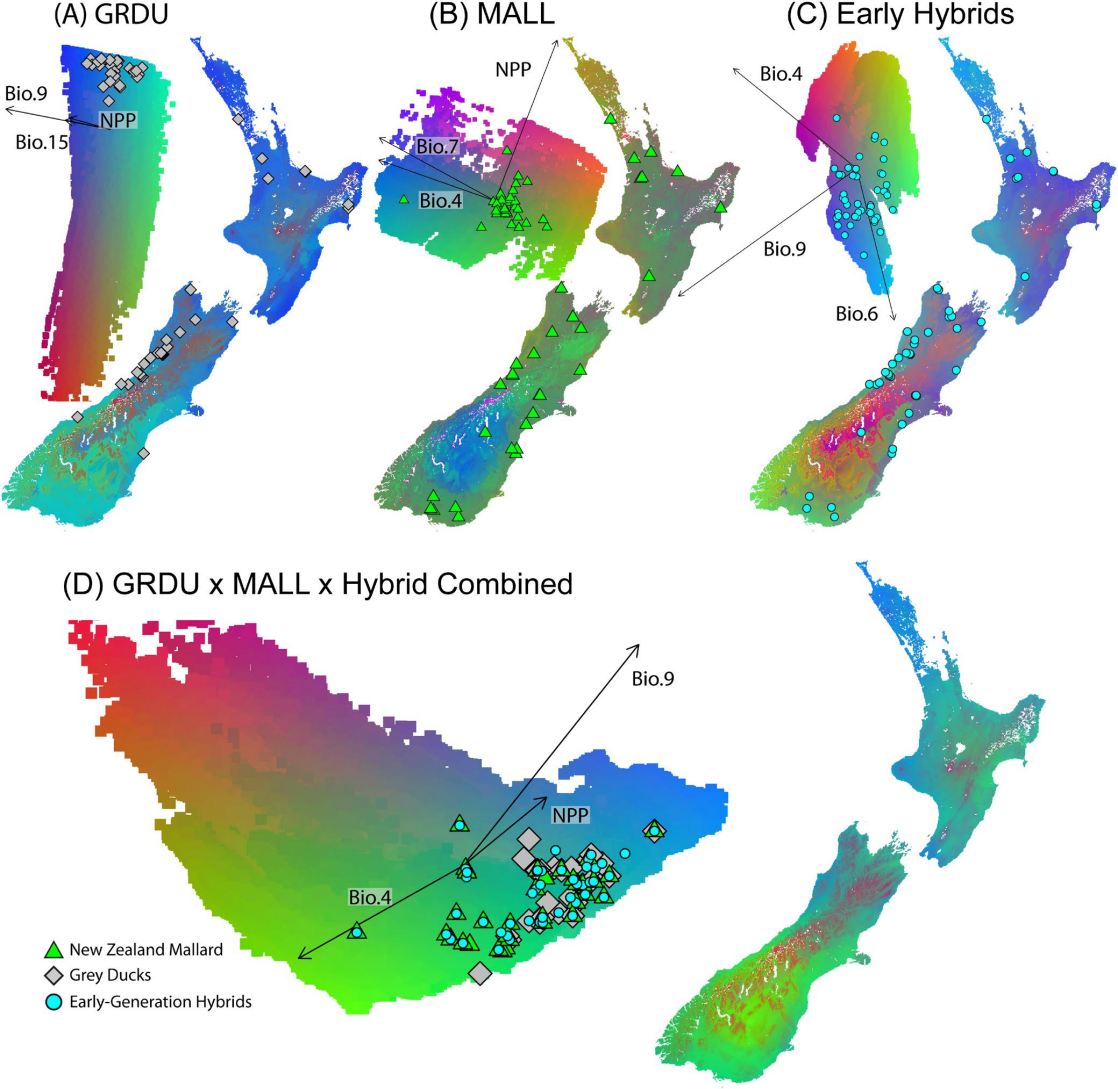
Genotype‐environment association models from gradientForest (GF) based on the top five most predictive environmental variables (Figure S5) mapped across NZ for (A) NZ Gray Ducks, (B) NZ Mallards, (C) early generation hybrids (F1–F3, including hybrid swarm individuals), and (D) the combined model. Note that GF models are unitless, and color changes represent expected changes in allele frequency in association with environmental clines. Insets represent PCA results from GF modeling.

Mapping PCs of GF outputs showed the Gray Duck's primary adaptive range to be somewhat limited to the mountainous regions of the western part of the South Island, with the majority of color changes (i.e., genotypic turnover) occurring outside of this range (Figure 4). We interpret overlap in areas with high Gray Duck ancestry and low genotypic turnover as indicative of adaptation to this region (Brown et al. 2022). Results for NZ Mallards and early‐generation hybrids were more variable and showed noticeable genotypic turnover across both islands (Figure 4). For Mallards, increasing genotypic turnover (i.e., a more rapid change in colors) corresponds with changes in ADMIXTURE frequencies across the landscape, particularly with the decrease in Mallard ancestry going from east to west across parts of both the North and South Islands. Early generation hybrids exhibited an intermediate pattern of genotypic turnover, demonstrating the adaptive influence from both parental lineages. The Gray Duck × Mallard combined model showed significant overlap in adaptive space, with Mallards notably expanding on the primary adaptive range of Gray Ducks (Figure S6). The Gray Duck × hybrid × Mallard and the Gray Duck × Mallard combined models generally reflect an adaptive refugia for Gray Ducks where genotypic turnover (i.e., change in color) is limited in their current remaining range. Alternatively, early‐generation hybrids inhabit large areas of genotypic turnover, encompassing the adaptive space of both parental lineages in the GF PCA (Figure 4; Figure S6).

**FIGURE 5:**
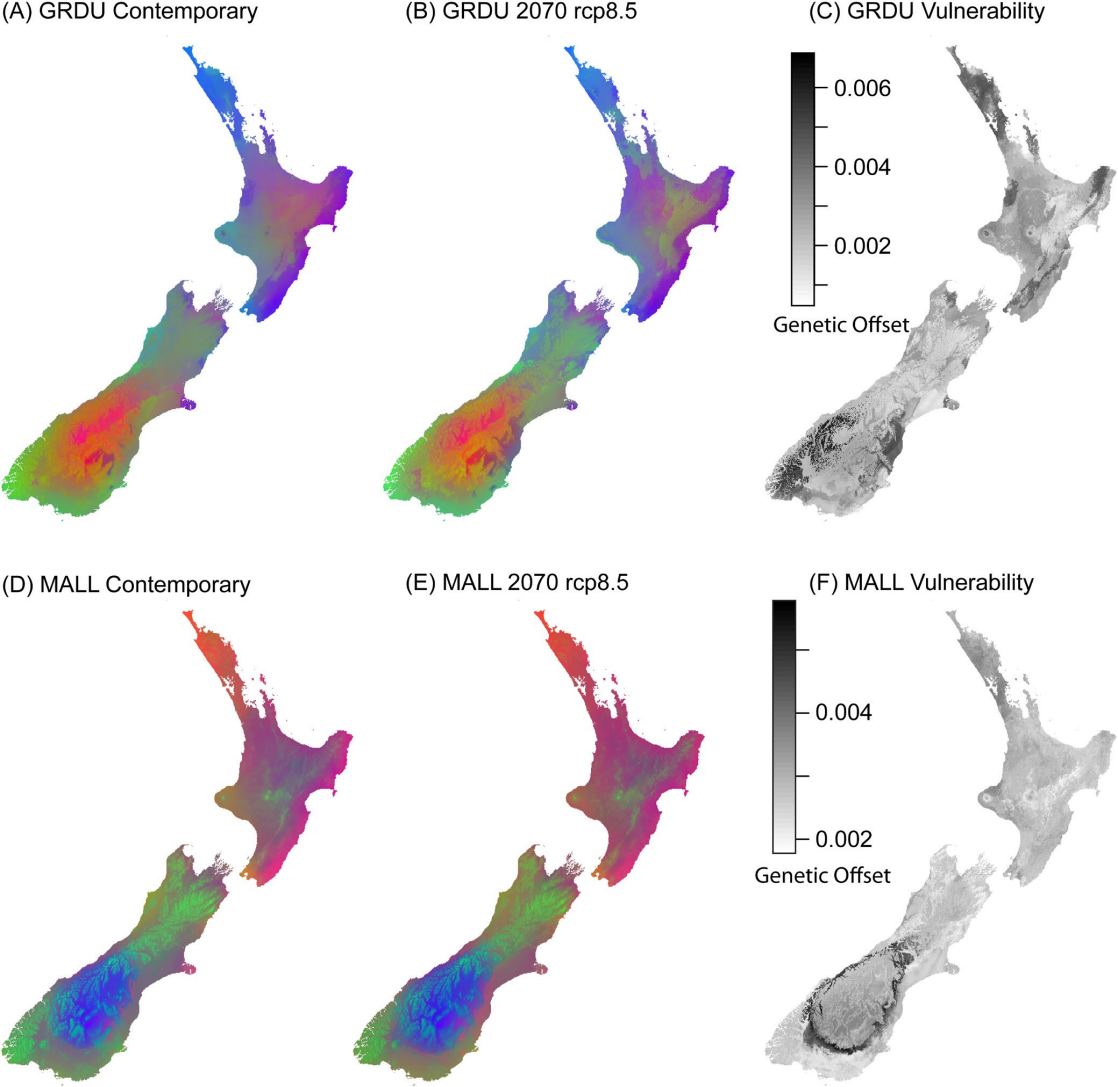
Contemporary (A) NZ Gray Duck and (D) NZ Mallard genotype‐environment association models from gradientForest (GF) based on the top five most predictive temperature and precipitation variables (Figure S5). (B, E) Associations are modeled across future environmental data for 2070 under the most extreme (rcp8.5) projections of climate change. Note that GF models (A, B, D, E) are unitless, and color changes represent expected changes in allele frequency. Finally, (C, F) Genomic offset calculated from the Euclidean distance between models based on contemporary and future climate conditions mapped across NZ.

Finally, genetic offset from future climate conditions (2070 rcp2.6 & rcp8.5) differed between Gray Ducks and Mallards. Gray Ducks were the more vulnerable group, as both the mild (rcp2.6) and extreme (rcp8.5) estimates of climate change showed large areas of genomic offset across most of NZ (Figure 5; Figure S7). Conversely, Mallard models under mild estimates of climate change showed limited offset from contemporary conditions (Figure S7), with areas of high vulnerability constrained to the arid mountain plateaus of both North and South Islands (Figure 5); however, Mallards showed widespread vulnerability similar to Gray Ducks under more extreme climate conditions (Figure S7).

## 4 Discussion

### 4.1 Population Genomics Reveals Remnant Gray Ducks Existing Alongside a Novel Mallard Hybrid Swarm in New Zealand

Taking a landscape genomics approach in NZ revealed that (1) isolated pockets of pure Gray Ducks still exist, while (2) the majority of samples collected represent a Gray Duck × NZ Mallard hybrid swarm, and that (3) Mallards introduced into NZ constitute a novel genetic cluster today (Figures 1 and 3; Peters et al. 2016). Despite expectations that Gray Ducks would have been genetically extinct by now (Gillespie 1985; Rhymer et al. 1994, 2004), pure Gray Ducks continue to persist (Figures 1 and 3), making up around 6% and 10% of all samples from the North and South Islands, respectively. These proportions are concordant with previous estimates from nearly four decades ago (Gillespie 1985; Rhymer et al. 1994, 2004), meaning that gene flow has stabilized and some form of reproductive isolation has prevented complete lineage fusion. Although the recovery of early‐generation hybrids (i.e., F1–F3; Figure 1, Figure S3) shows that hybridization continues, their limited numbers (Figures 2B and 3) suggest that interspecific pairings are rare overall today. Instead, the overwhelming number of undefined hybrids supports a hybrid swarm scenario in which admixed individuals are primarily the result of hybrid × hybrid pairings (Culumber et al. 2014).

While genetic drift has undoubtedly influenced the contemporary genetic diversity of NZ Mallards, there has been disproportionate introgression of novel genetic variation from Gray Ducks (Figure 1A). This appears to at least in part explain why NZ Mallards show such strong genomic differentiation from their parental Mallard stock (i.e., game‐farm and North American wild Mallards) and are genetically intermediate to Gray Ducks in PCA space (Figures 1 and 3; Figure S2). Moreover, the combined GF models show that NZ Mallards' adaptive space encompasses and expands on that of native Gray Ducks (Figure S7). Indeed, NZ Mallards represent a thriving self‐sustaining population of nearly 3 million (McDougall and Amundson 2017), with survival and breeding metrics comparable to wild North American populations (Sheppard 2017; Sheppard et al. 2019). Together, we conclude that extensive gene flow has indeed resulted in the genetic swamping (Sheppard 2017; Sheppard et al. 2019) of the original introduced Mallard stock, and that NZ Mallards now constitute a hybrid swarm. In general, NZ Mallards demonstrate the role of strong selective pressures, introgression, and genetic drift in promoting local adaptation, begging the question of whether this once domestically‐derived lineage may now be a uniquely adapted evolutionary unit? Further research into population vital rates and high‐resolution whole‐genome sequencing is needed to further answer these questions.

### 4.2 Human‐Induced Breakdown of Reproductive Barriers and Introgression Facilitating Non‐Native Species Expansion

Anthropogenic hybridization is often variable across the landscape, as heterogeneous selective pressures are artificially influenced by changes in land use and habitat degradation (Foley et al. 2005). This type of transformation can lead to a breakdown of reproductive isolation in some areas as anthropogenic changes interrupt selective pressures that drive natural behaviors (Malukiewicz et al. 2015). Variable rates of hybridization among Gray Ducks and Mallards across NZ suggest that any reproductive isolation that may have existed has broken down in areas where the two species co‐occur. Following their primary introduction nearly a century ago, the expansion of Mallards directly followed the replacement of native NZ wetlands and forests with farmland (Taylor and Dizon 1999; Holdaway et al. 2012) and the current drastic decline of Gray Ducks (Balham 1952). Such disturbances likely acted to facilitate hybridization, as areas with the most severe habitat degradation generally overlap areas where the hybrid swarm is now concentrated (e.g., the eastern coasts of the South and North Islands; Figure 2A). In fact, the genotypic turnover in the combined Gray Duck × Mallard GF model not only recapitulates the putative range of the remaining Gray Duck population but also identifies it as distinct niche space partitioned from areas dominated by Mallards and hybrids (i.e., the eastern parts of the South and North Islands; Figure 4; Figure S6). Overall, this suggests that the timing of Mallard introductions following human‐induced habitat changes contributed to the quick establishment of feral Mallard populations throughout NZ.

While the theoretical complexities of biological invasions have been widely discussed, empirical evidence on the roles that various evolutionary mechanisms play in this process is lacking (Davis and Thompson 2000; Richardson et al. 2000; Colautti and MacIsaac 2004; Valéry et al. 2008). Moreover, invasion consists of two separate processes—establishment and expansion—that are differentially affected by the strength of selective and neutral processes (i.e., genetic drift), as well as levels of gene flow with closely related native taxa (Drake 2006). We contend that the NZ Mallard population provides an important wild study system in which many of these complex processes appear to be interacting. Whereas the original NZ Mallards primarily consisted of domestic and wild stock from North America, we posit that extensive introgression from native Gray Ducks likely enabled a rapid 100‐fold population increase of NZ Mallards over the last half century. More specifically, in a kind of ‘catapult’ effect, habitat modification and overharvesting of Gray Ducks combined with the introduction of novel genetic variation is a plausible explanation regarding the initial establishment and expansion of NZ Mallards (Verhoeven et al. 2011). In fact, this hypothesis is supported in the combined GEA models that identify contemporary hybrids, including NZ Mallard and Gray Duck backcrosses with an increased adaptive range that encompasses either Gray Ducks or NZ Mallards on their own (Figure 4). In general, this reaffirms the important role that adaptive gene flow can play during the establishment phase of biological invasions (Keller and Taylor 2010; Verhoeven et al. 2011). Although we continue to demonstrate the utility of reduced‐representation sequencing data when used with landscape‐level sample sets, future research will benefit from whole‐genome re‐sequencing, which will be necessary to determine whether the rapid establishment of NZ Mallards could be a case of adaptive introgression.

### 4.3 Impact of Climate Change and Conservation in New Zealand

Understanding the relationship between genotype and the environment has become a major tool for evaluating the vulnerability of natural populations under future climate change scenarios (Owens and Samuk 2020; Brown et al. 2022). Given that many invasive taxa could stand to thrive due to climate change (Macinnis‐Ng et al. 2021), GEA methods can be employed to predict the adaptive potential of such taxa under future environments, as well as identify areas where expansion might further threaten local populations. Our GEA models identify Gray Ducks as at least moderately vulnerable to climate change, regardless of the severity of the climate model (Figure 5). Interestingly, the eastern and southeastern regions of the North and South Islands, where pure Gray Ducks are already rare, showed the most significant genetic offset in our models (Figure 5). This suggests that these regions are potentially already unsuitable as Gray Ducks were absent from the eastern side of the Southern Alps, with the exception of a single individual (Figure 5; McGlone 2009). Ecologically, this means that Gray Ducks persist in areas of more undisturbed habitat, such as the higher elevation mountainous streams, lakes, and tidal estuaries that make up the western coasts and southern portion of the Southern Alps (Figure 2; Williams 1964). Unfortunately, GEA models also predict that these remaining core habitats could be threatened due to climate change (Figure 5). Even if high elevation wetlands provide limited refugia along the west coast, the movement of Gray Ducks out of their current range will likely lead to further breakdown of ecological partitioning with NZ Mallards and hybrids, making lineage fusion more probable. Alternatively, if geography (i.e., Cook strait and the Southern Alps) and not ecological partitioning is acting as a primary reproductive barrier, then Gray Ducks may be able to persist as NZ Mallards reportedly travel only short distances (< 25 km; Caithness et al. 1991; Mcdougall 2012); although occasional vagrants have been re‐captured as far as Norfolk Island and New Caledonia (pers. comm. M. McDougall, Eastern fish and Game and D. Klee, Auckland/Waikat Fish and Game). Overall, native wetland restoration across NZ, which would strengthen existing reproductive barriers by increasing suitable habitat for Gray Ducks, would likely be the most substantial conservation strategy.

Unlike the Gray Duck, GEA models of genomic vulnerability for the NZ Mallard were milder (Figure 5). For the more moderate climate scenarios (rcp2.6), NZ Mallards showed very little genetic offset to contemporary models (Figure S7), suggesting that they could be more resilient to future climate change. However, considering the most extreme climatic scenarios (rcp8.5), there is strong potential for NZ Mallards and hybrids to become maladaptive across the North Island, similar to Gray Ducks (Figure 5). Although incorporating standing genetic variation allows us to better estimate adaptive potential, GF models cannot account for land use change in the future. Approximately 90% of the native wetlands and 60% of all land cover throughout NZ have already been converted for agricultural purposes or urban development (MacLeod and Moller 2006; Ausseil et al. 2011; Myers et al. 2013); as this trend continues, NZ Mallards would be the most likely to benefit, as they are known to take advantage of small wetlands within these agricultural and high‐disturbance anthropogenic areas (Figley and VanDruff 1982; English et al. 2017).

### 4.4 Considerations of Adaptive Potential and Conservation in the Anthropocene

While the main principle of conservation biology is to preserve biodiversity and ecosystem functions, specific conservation goals are not always congruent across political or organizational boundaries (Dallimer and Strange 2015). This has prompted increased discussion on how anthropogenic hybridization in an era of rapid environmental change should be viewed (Hirashiki et al. 2021). Although introgression can enhance genetic diversity by providing advantages for adaptation under future environmental conditions, conservation efforts should not prioritize this benefit at the expense of preserving locally native species. We conclude that NZ's Mallard‐like ducks provide a cautionary case study where anthropogenic hybridization coupled with excessive landscape changes and habitat loss has likely contributed to the naturalization of an introduced population at the expense of native species. In this case, Gray Ducks are no longer able to compete with their introduced counterparts, which are now potentially better adapted to an increasingly anthropogenic landscape. We caution that the complex ecological effects originating from such extensive introgression are unpredictable and can easily break the equilibrium between selection and gene flow that has thus far acted to maintain at least some of the original diversity from both parental taxa. Therefore, the conservation of Gray Ducks should remain a priority in NZ, as extensive introgression is ongoing and likely contributing to their own demise.

In addition to the challenges faced by Gray Ducks, the broader ecological and cultural roles played by introduced populations such as the NZ Mallards should also be considered when deciding their fate, as introgression from native taxa can often provide traits necessary to thrive. Despite their primarily domestic origin, NZ Mallards have experienced high levels of gene flow and strong selective pressures from their novel environment for nearly a century, resulting in a genetically and phenotypically distinct Mallard that no longer resembles its parental populations in terms of its genetic lineage (Figure 1) or adaptive space (Figure 5). Moreover, stable population trends (McDougall and Amundson 2017) along with survival and fecundity rates that are comparable to other wild populations (Sheppard 2017) suggest that NZ Mallards potentially constitute a case of human‐induced naturalization. While the adaptive advantages of introduced and/or hybrid individuals require careful evaluation on a case‐by‐case basis, native Gray Duck habitat restoration should remain a priority as the negative effects of introgression may yet be fully realized (Tufto 2017).

## Author Contributions

**Joshua I. Brown:** conceptualization (equal), data curation (equal), formal analysis (equal), funding acquisition (equal), writing – original draft (equal). **Jennifer L. Sheppard:** conceptualization (equal), data curation (equal), writing – original draft (equal). **Jonathon Mohl:** data curation (equal), writing – original draft (equal). **Irene E. Engilis:** data curation (equal), funding acquisition (equal), writing – original draft (equal). **Andrew Engilis Jr.:** conceptualization (equal), funding acquisition (equal), writing – original draft (equal). **Philip Lavretsky:** conceptualization (equal), data curation (equal), formal analysis (equal), funding acquisition (equal), writing – original draft (equal).

## Conflicts of Interest

The authors declare no conflicts of interest.

## Supporting information


**Table S1.** Individual sample and locale information.

**Table S2.** List of environmental variables used for genotype‐environment association testing in gradientForest.
**Table S3.** Assignment probabilities from ADMIXTURE‐based simulation for hybrid backcrosses into both parental species (F1—F10).
**Figure S1.** Sequencing depth for Z and W‐chromosome ddRAD‐seq loci used to determine sex for each sample.
**Figure S2.** Composite Φ_ST_ estimates for Autosomal, Z‐chromosome, and mtDNA loci (NI = North Island, SI = South Island).
**Figure S3.** (A) The average and range of assignment probabilities from ADMIXTURE results at *K* of 2 and 3 across 25 simulated replications of hybridization (F1) and nine generations of backcrossing (F2–F10) using genetically vetted Gray Ducks (GRDU) and NZ Mallards (MALL) – each *K* is based on 250 independent ADMIXTURE analyses. Simulations established assignment probability bins for parental Gray Ducks, Mallards, F1 hybrids, three (F2‐GRDU/MALL, F3‐GRDU/MALL & F4‐ABDU/MALL) categories for Gray Duck or NZ Mallard‐backcrosses (also see Tables S1 and S2). Empirical assignment probabilities of samples obtained from the (B) North or (C) South Islands are provided.
**Figure S4.** A haplotype network based on 600 base‐pairs of the mitochondrial control region and sequenced for NZ and reference North American wild and game‐farm Mallards. NZ samples are color coded by their nuclear assignment (Figure S3) to parental Gray Duck, NZ Mallard (both South and North Island Mallards were grouped here), F1 hybrids, and various backcrossed generations.
**Figure S5.** Cumulative *R* ^2^ weighted importance ranking of 27 environmental predictor variables from gradientForest.
**Figure S6.** Combined model of genotype‐environment associations from gradientForest for NZ Gray Ducks and NZ Mallards projected across NZ.
**Figure S7.** Contemporary (A) NZ Gray Duck and (D) NZ Mallard genotype‐environment association models from gradientForest (GF) based on the top five most predictive temperature and precipitation variables (Figure S5). (B, E) Associations are modeled across future environmental data for 2070 under the mildest (rcp2.6) projections of climate change. Note that GF models (A, B, D, E) are unitless, and changes in color represent expected changes in allele frequency. Finally, (C, F) Genomic offset calculated from the Euclidean distance between models based on contemporary and future climate conditions mapped across NZ.

## Data Availability

BioProject: PRJNA1238238; PRJNA1106373; PRJNA980669. GenBank ddRAD Raw Reads Accessions: SAMN35641155—SAMN47463276. GenBank mtDNA Accessions: PP746861–PP746942; PV346099–PV346671; OR89157–OR90543.
