# Supplementary material for: Anthropogenic Hybridization Contributes to the Naturalization of Introduced Domestic Mallards (Anas platyrhynchos) at the Expense of Native New Zealand Gray Ducks (A. s. superciliosa)
Source: Ecol Evol. 2025 Jun 7;15(6):e71536. doi: 10.1002/ece3.71536 (PMC12144591; doi:10.1002/ece3.71536)

**SUPPLEMENTARY MATERIALS**

**TITLE: Anthropogenic Hybridization Contributes to the Naturalization of Introduced Domestic Mallards (*Anas platyrhynchos*) at the Expense of Native New Zealand Grey Ducks (*A. s. superciliosa*)**

**Running Title: Naturalization of domestic mallards**

**Table S2.** List of environmental variables used for genotype-environment association testing in GradientForest.

| Variable | Description | Unit |
| --- | --- | --- |
| BIO1 | Mean annual temperature | °C |
| BIO2 | Mean diurnal range | °C |
| BIO3 | Isothermality | °C |
| BIO4 | Temperature seasonality | °C |
| BIO5 | Max temperature of warmest month | °C |
| BIO6 | Min temperature of coldest month | °C |
| BIO7 | Temperature annual range | °C |
| BIO8 | Mean temperature of wettest quarter | °C |
| BIO9 | Mean temperature of driest quarter | °C |
| BIO10 | Mean temperature of warmest quarter | °C |
| BIO11 | Mean temperature of coldest quarter | °C |
| BIO12 | Annual precipitation | mm |
| BIO13 | Precipitation of wettest month | mm |
| BIO14 | Precipitation of driest month | mm |
| BIO15 | Precipitation seasonality | mm |
| BIO16 | Precipitation of wettest quarter | mm |
| BIO17 | Precipitation of driest quarter | mm |
| BIO18 | Precipitation of warmest quarter | mm |
| BIO19 | Precipitation of coldest quarter | mm |
| NDVI_ANNUAL | Normalized Difference Vegetation Index Annual; MOD13A3 |  |
| NDVI_WINTER | Normalized Difference Vegetation Index June; MOD13A3 |  |
| NDVI_SUMMER | Normalized Difference Vegetation Index December; MOD13A3 |  |
| EVI_ANNUAL | Enhanced Vegetation Index Annual; MOD13A3 |  |
| EVI_WINTER | Enhanced Vegetation Index June; MOD13A3 |  |
| EVI_SUMMER | Enhanced Vegetation Index December; MOD13A3 |  |
| NPP_ANNUAL | Net Primary Productivity Annual; MOD17A2H |  |
| SRTM | Shuttle radar topography mission; Elevation | m |

**Table S3**. Assignment probabilities from ADMIXTURE based simulation for hybrid backcrosses into both parental species (F1 – F10).


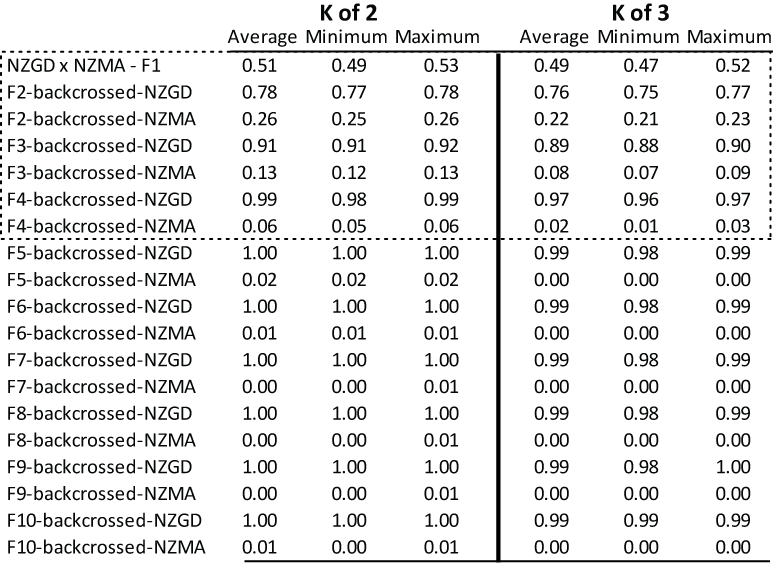


**Figure S1**. Sequencing depth for Z and W-chromosome ddRAD-seq loci used to determine sex for each sample.

**
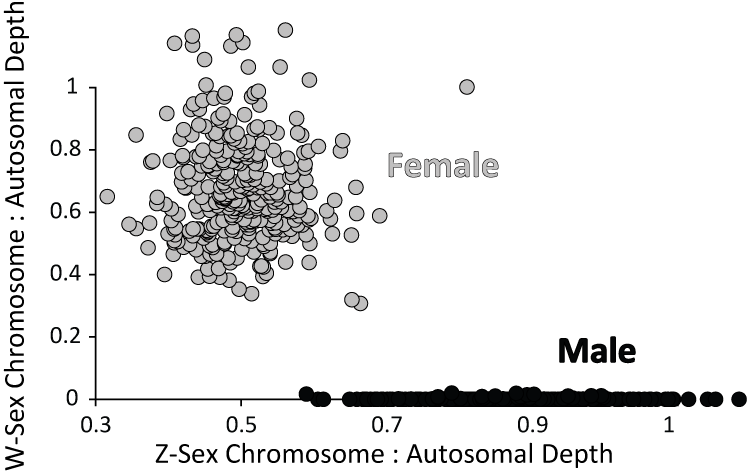
**

**Figure S2**. Composite Φ_ST_ estimates for Autosomal, Z-chromosome, and mtDNA loci (NI = North island, SI = South island).

**
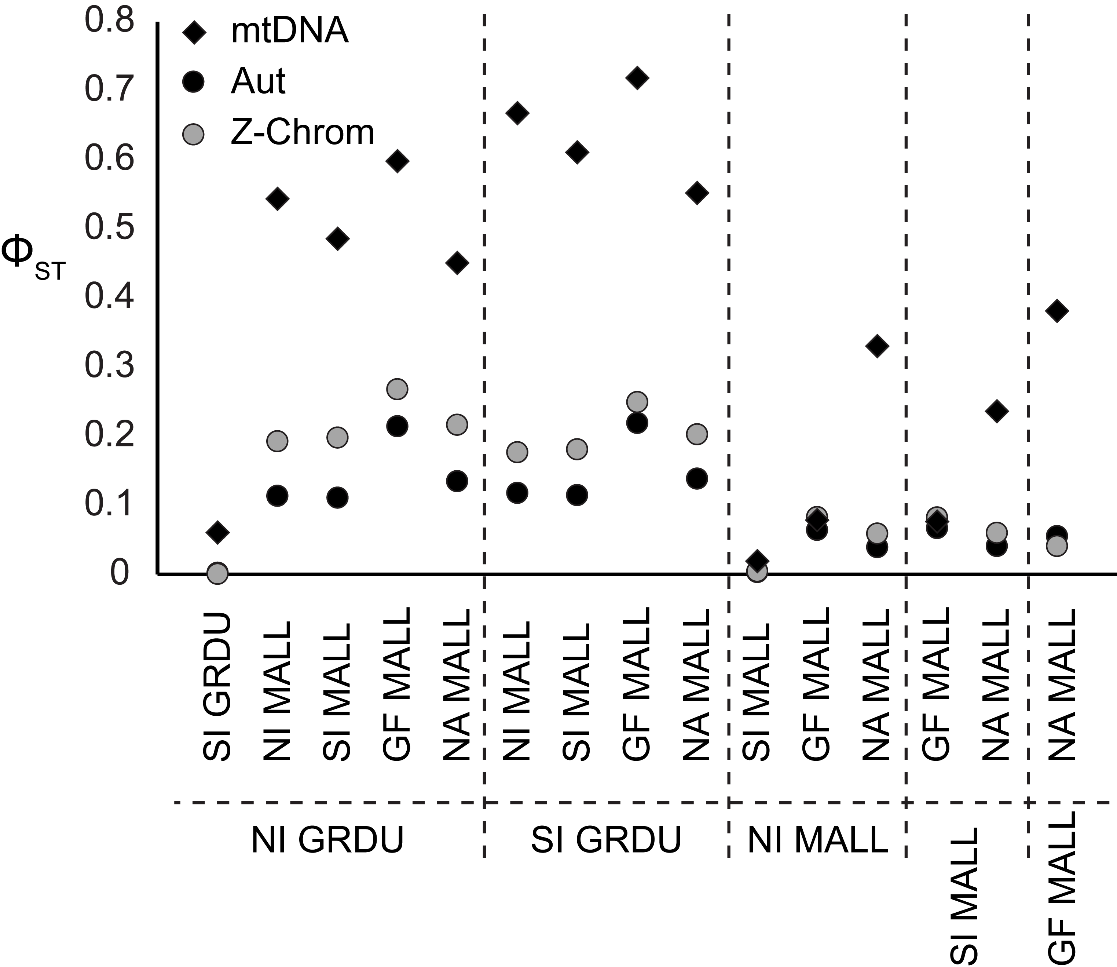
**

**Figure S3.** (A) The average and range of assignment probabilities from ADMIXTURE results at *K* of 2 and 3 across 25 simulated replications of hybridization (F1) and nine generations of backcrossing (F2-F10) using genetically vetted grey ducks (GRDU) and NZ mallards (MALL) – each *K* is based on 250 independent ADMIXTURE analyses. Simulations established assignment probability bins for parental grey ducks, mallards, F1 hybrids, three (F2-GRDU/MALL, F3-GRDU/MALL & F4-ABDU/MALL) categories for grey duck or NZ mallard-backcrosses (also see Supplementary Materials Tables S1 & S2). Empirical assignment probabilities of samples obtained from the (B) North or (C) South Islands are provided.

**
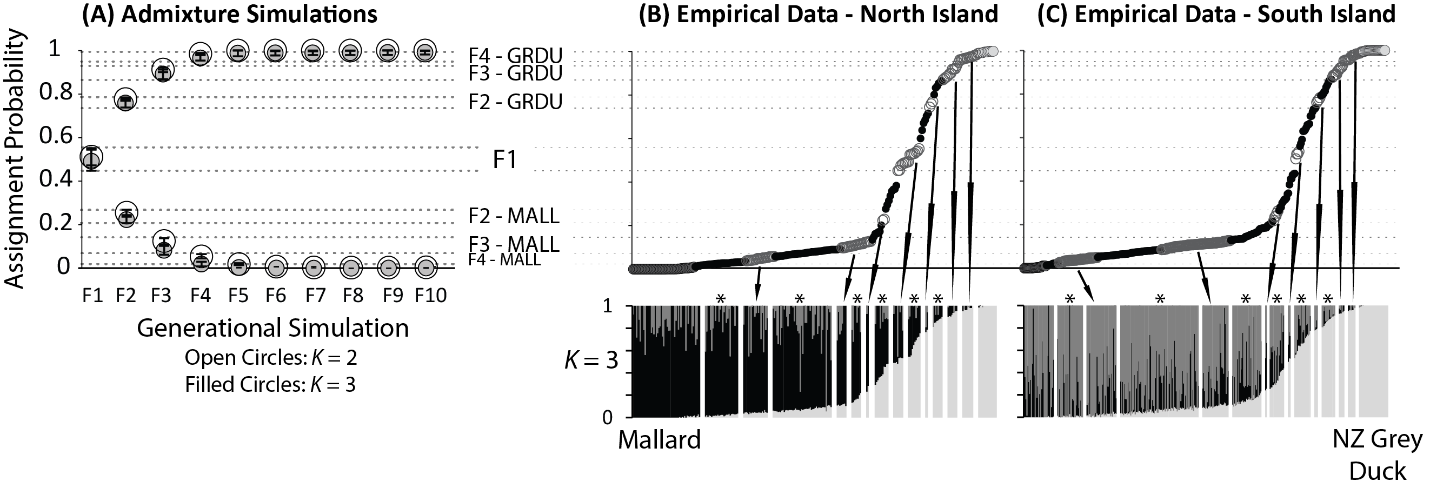
**

**Figure S4.** A haplotype network based on 600 base-pairs of the mitochondrial control region and sequenced for New Zealand and reference North American wild and game-farm mallards. New Zealand samples are color coded by their nuclear assignment (Supplementary Materials Figure S3) to parental grey duck, New Zealand mallard (both South and North Island mallards were grouped here), F1 hybrids, and various backcrossed generations.

**
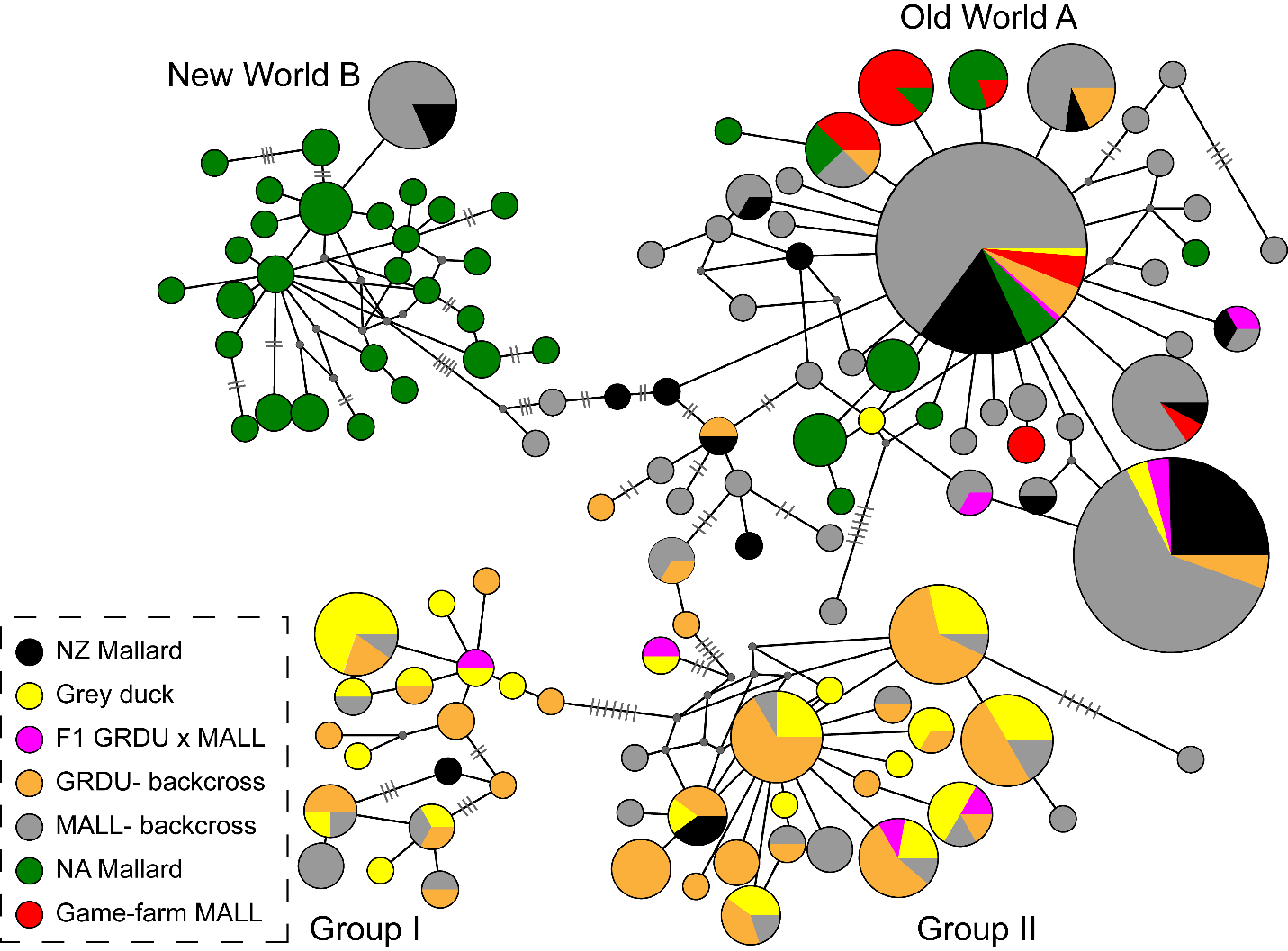
**

**Figure S5**. Cumulative R^2^ weighted importance ranking of 27 environmental predictor variables from gradientForest.

**
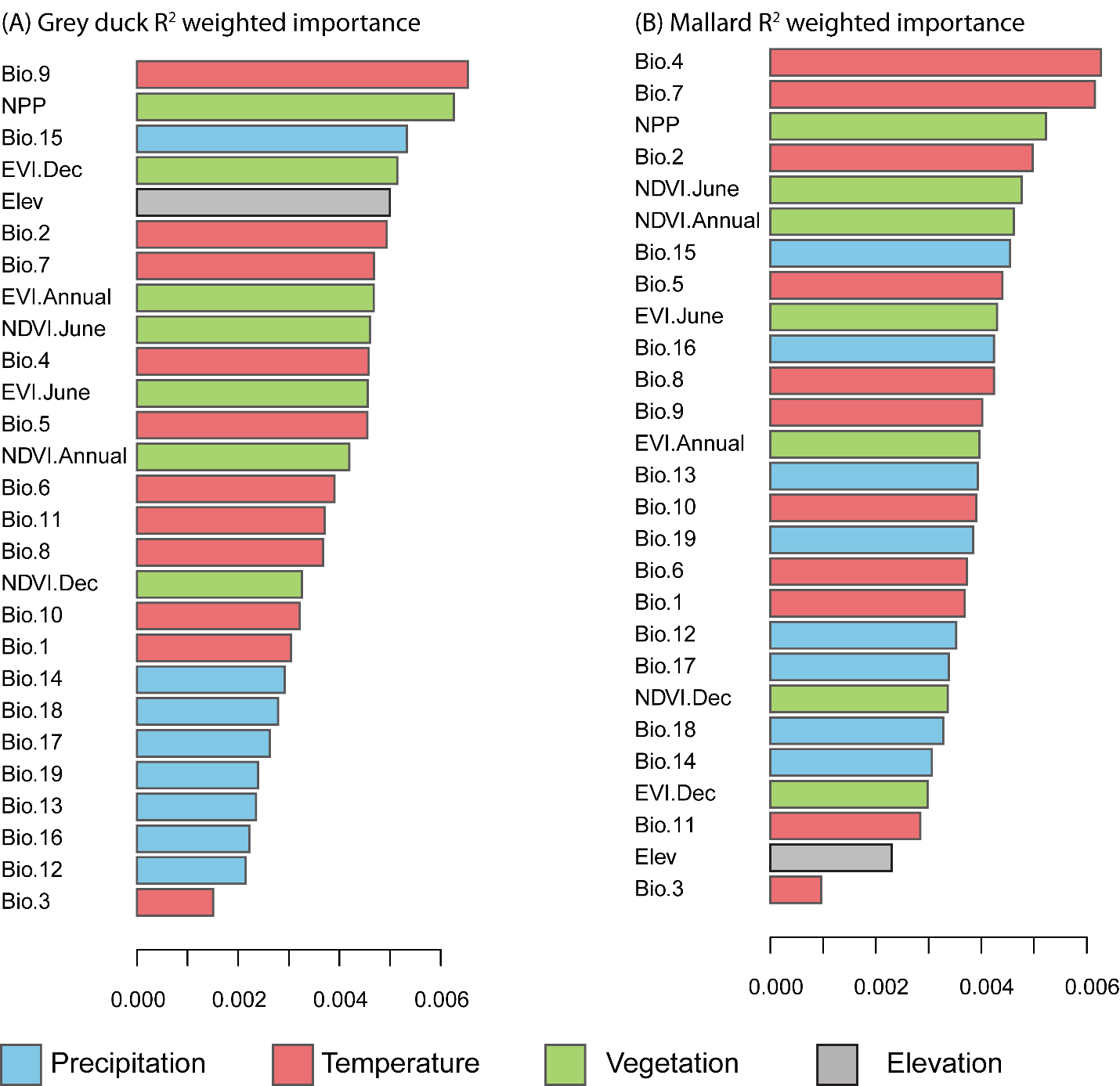
**

**Figure S6**. Combined model of genotype-environment associations from gradientForest for New Zealand grey ducks and New Zealand mallards projected across New Zealand.

**
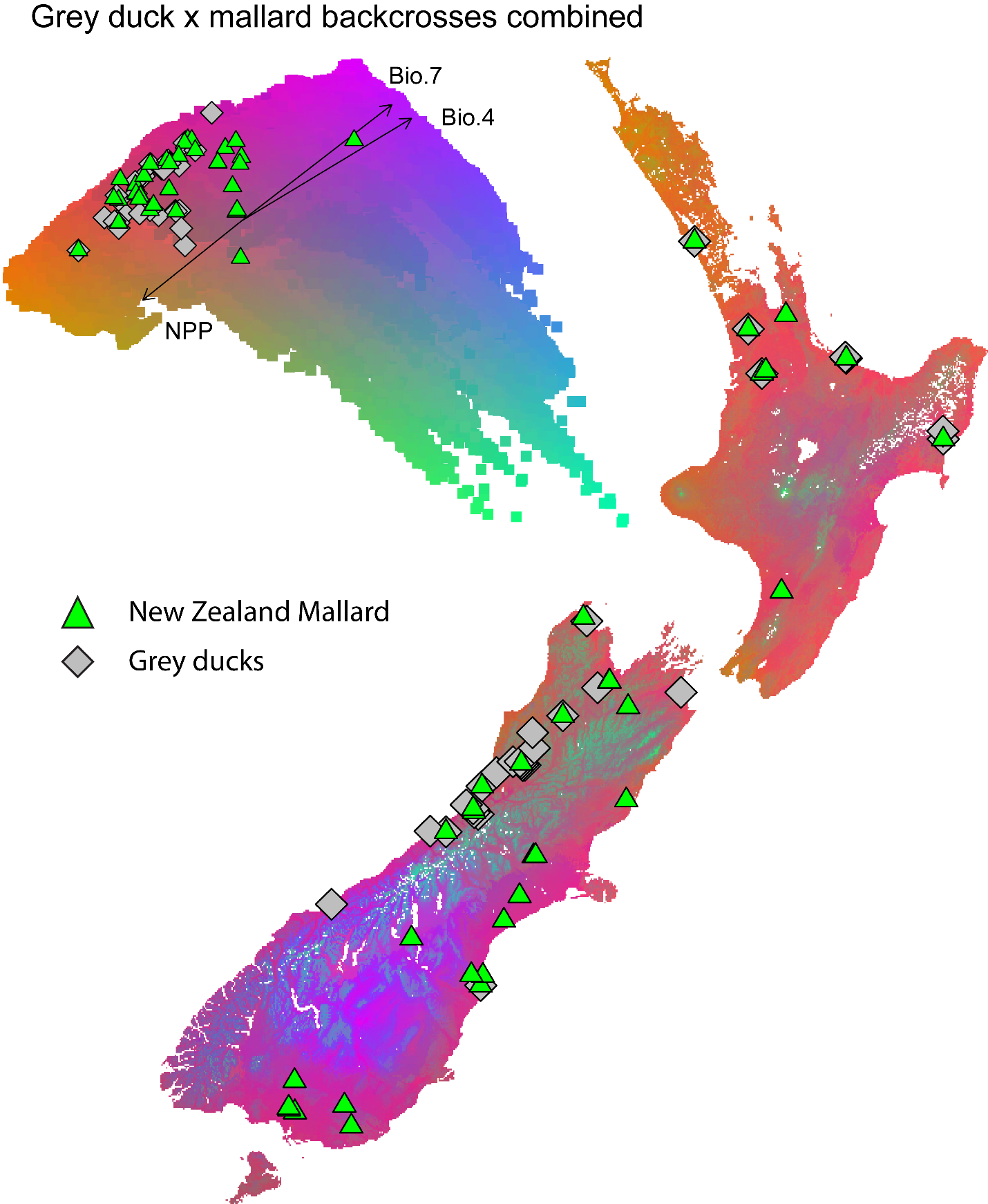
**

**Figure S7.** Contemporary (A) New Zealand grey duck and (D) New Zealand mallard genotype-environment association models from gradientForest (GF) based on the top five most predictive temperature and precipitation variables (Supplementary Materials Figure S5). (B, E) Associations are modelled across future environmental data for 2070 under the mildest (rcp2.6) projections of climate change. Note that GF models (A, B, D, E) are unitless, and changes in color represent expected changes in allele frequency. Finally, (C, F) Genomic offset calculated from the Euclidean distance between models based on contemporary and future climate conditions mapped across New Zealand.


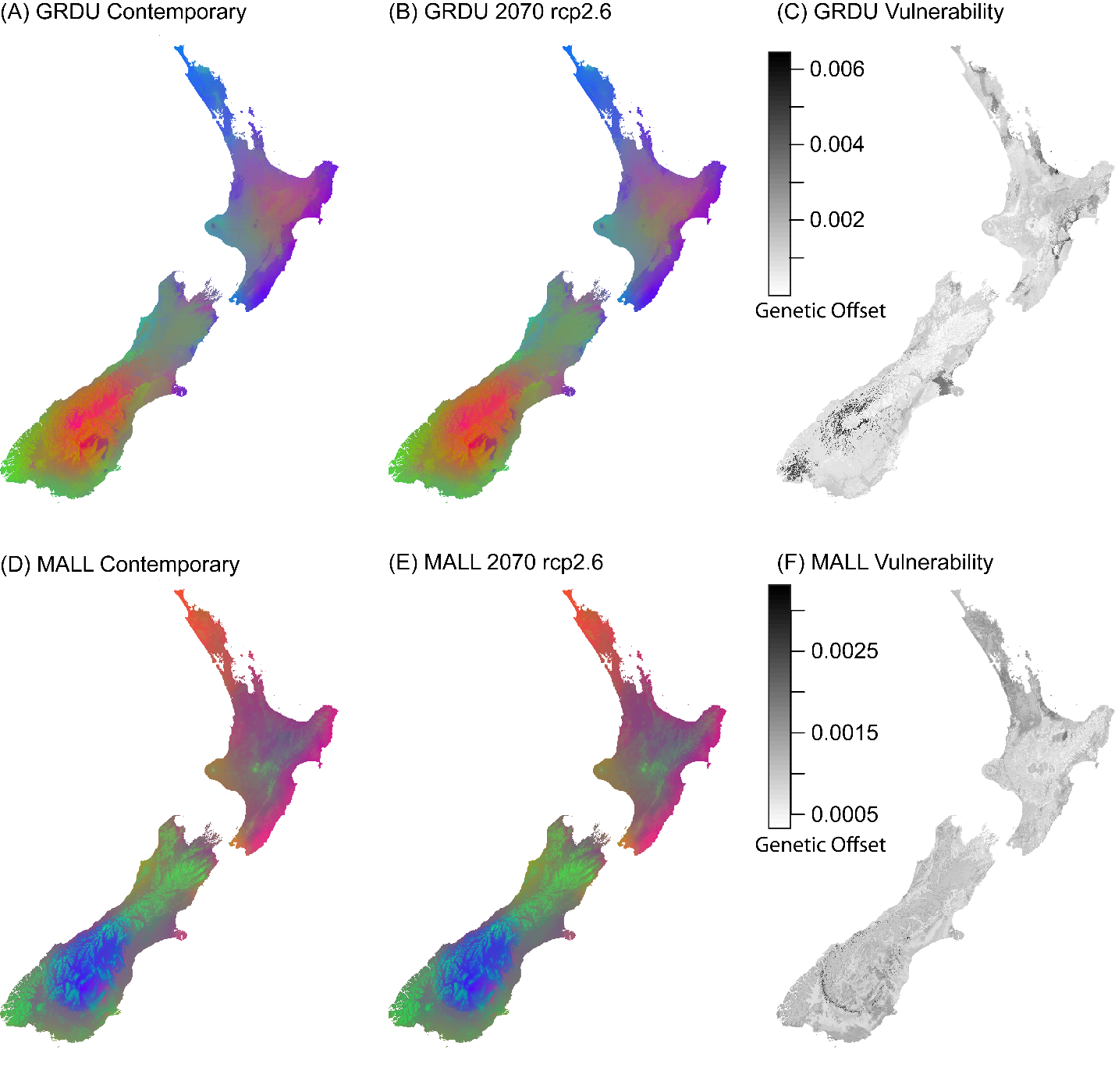

Supplement: Supplementary file 2 — Table S2. List of environmental variables used for genotype‐environment association testing in gradientForest. Table S3. Assignment probabilities from ADMIXTURE‐based simulation for hybrid backcrosses into both parental species (F1—F10). Figure S1. Sequencing depth for Z and W‐chromosome ddRAD‐seq loci used to determine sex for each sample. Figure S2. Composite ΦST estimates for Autosomal, Z‐chromosome, and mtDNA loci (NI = North Island, SI = South Island). Figure S3. (A) The average and range of assignment probabilities from ADMIXTURE results at K of 2 and 3 across 25 simulated replications of hybridization (F1) and nine generations of backcrossing (F2–F10) using genetically vetted Gray Ducks (GRDU) and NZ Mallards (MALL) – each K is based on 250 independent ADMIXTURE analyses. Simulations established assignment probability bins for parental Gray Ducks, Mallards, F1 hybrids, three (F2‐GRDU/MALL, F3‐GRDU/MALL & F4‐ABDU/MALL) categories for Gray Duck or NZ Mallard‐backcrosses (also see Tables S1 and S2). Empirical assignment probabilities of samples obtained from the (B) North or (C) South Islands are provided. Figure S4. A haplotype network based on 600 base‐pairs of the mitochondrial control region and sequenced for NZ and reference North American wild and game‐farm Mallards. NZ samples are color coded by their nuclear assignment (Figure S3) to parental Gray Duck, NZ Mallard (both South and North Island Mallards were grouped here), F1 hybrids, and various backcrossed generations. Figure S5. Cumulative R 2 weighted importance ranking of 27 environmental predictor variables from gradientForest. Figure S6. Combined model of genotype‐environment associations from gradientForest for NZ Gray Ducks and NZ Mallards projected across NZ. Figure S7. Contemporary (A) NZ Gray Duck and (D) NZ Mallard genotype‐environment association models from gradientForest (GF) based on the top five most predictive temperature and precipitation variables (Figure S5). (B, E) Associations are m [file ECE3-15-e71536-s001.docx]
